# Investigating the cardioprotective effects of Fuzheng Yangxin recipe based on network pharmacology and experimental evaluation

**DOI:** 10.3389/fphar.2022.1004929

**Published:** 2022-09-26

**Authors:** Anzhu Wang, Wei Zhao, Kaituo Yan, Lijun Guo, Feng Gao, Jingjing Chen, Yifei Wang, Xiaochang Ma

**Affiliations:** ^1^ Xiyuan Hospital, China Academy of Chinese Medical Sciences, Beijing, China; ^2^ Graduate School, China Academy of Chinese Medical Sciences, Beijing, China; ^3^ Yidu Central Hospital of Weifang, Weifang, China; ^4^ National Clinical Research Center for Chinese Medicine Cardiology, Beijing, China; ^5^ Beijing University of Chinese Medicine, Beijing, China

**Keywords:** ischemic heart disease, heart failure, Fuzheng Yangxin recipe, network pharmacology, bioinformatics analysis, experimental verification

## Abstract

**Background:** Under Chinese medicine theory guidance, Fuzheng Yangxin Recipe (FZYX) is clinically effective for the treatment of heart failure (HF) caused by ischemic heart disease (IHD). This study aimed to investigate the mechanism of the myocardial protective effects of FZYX on HF.

**Materials and methods:** The Gene expression omnibus database was used to identify differential genes of the IHD subtype. Through network pharmacological methods, the targets of the active components of FZYX were obtained. We also constructed IHD-induced HF model rats by ligating the left anterior descending coronary artery. Echocardiography, pathological section staining, enzyme-linked immunosorbent assay, western blotting, immunohistochemistry, and quantitative real-time PCR analyses were performed to verify the protective effects of FZYX on the myocardium.

**Results:** We identified 53 active components and 37 potential targets of FZYX associated with the IHD subtype. Signal transducer and activator of transcription 3 (STAT3) is a key protein in the protein-protein interaction (PPI) network. A total of 146 biological processes, 10 cellular components and 40 molecular function subcategories were identified by Gene Ontology (GO) enrichment analysis, and 18 signalling pathways, including apoptosis, were identified by Kyoto Encyclopedia of Genes and Genomes (KEGG) enrichment analysis. *In vivo* experiments showed that FZYX significantly inhibited cardiomyocyte apoptosis, promoted the expression and phosphorylation of STAT3, and improved cardiac function.

**Conclusion:** FZXY improves cardiac function and protects cardiomyocytes from injury via multi-component, multi-target and multi-pathway action, especially its possible role in regulating STAT3 expression and anti-apoptotic effect.

## 1 Introduction

Heart failure (HF) is one of the main public health problems worldwide. In the 2017 Global Burden of Disease Study, the number of HF patients was 64.34 million (Disease, 2018). Data from the USA in 2019 showed that 6.2 million people aged >20 years suffered HF, and HF-associated morbidity is estimated to increase by 46% from 2012 to 2030 (Benjamin et al., 2019). Ischemic heart disease (IHD) is an important cause of HF, and acute myocardial infarction (MI) is often the first manifestation. The China-HF Registry indicates that patients with HF and coronary heart disease account for 49.6% (Zhang et al., 2017). With the advancement of medical treatment, the morbidity of HF following MI has decreased, but the mortality is still high. A SWEDEHEART study involving 199,851 cases of acute MI showed that the incidence of HF after acute MI dropped from 46% in 1996 to 28% in 2008. However, the 1-year mortality after HF only dropped from 36% in 1996 to 31% in 2008, which is higher than in non-HF patients (Desta et al., 2015). This is because although reperfusion therapy can save the ischemic myocardium caused by infarction, it can also induce other irreversible damage such as cardiomyocyte necrosis and coronary microvascular dysfunction, which may aggravate ventricular remodelling (Heusch, 2020). Therefore, drug treatment of HF caused by IHD is a major research focus.

HF is not mentioned in traditional Chinese medicine (TCM), but its clinical symptoms were described more than 2000 years ago. Chinese medicine proposes that the onset of HF is related to impairment of the “*Yangqi*” in the heart, and herbal medicine is widely used in the treatment of patients with HF in China (Tang and Huang, 2013). Fuzheng Yangxin Recipe (FZYX) is a TCM concoction consisting of 6 g of *Panax notoginseng (Burkill) F. H. Chen*, 20 g of *Panax ginseng C. A. Meyer*, 30 g of *Rhodiola crenulata (Hook.f.et Thoms.) H. Ohba*, 10 g of *Aconitum carmichaeli Debx*, 20 g of *Ophiopogon japonicus (Linn. f.) Ker-Gawl*, 30 g of *Astragalus membranaceus (Fisch.) Bunge*, 15 g of *Angelica sinensis (Oliv.) Diels* and 15 g of *Rehmannia glutinosa Libosch*. In clinical practice, FZYX has a beneficial effect on patients with HF caused by IHD (Guo, 2018; Zheng, 2020), but the underlying mechanism remains unclear.

Network pharmacology, based on systems biology and multi-directional pharmacology, allows the selection of specific nodes using biomolecular network analysis methods to carry out molecular design and target analysis of drugs (Gosak et al., 2018). This method is dynamic, systemic and interactive, and can construct a ‘disease-gene-target-component-drug’ network from the perspective of systems biology, which is highly coincident with the holistic concept, and syndrome differentiation and treatment of TCM(Kibble et al., 2015). The present study explored the mechanism of FZYX in the treatment of HF based on network pharmacology, including *in vivo* experiments. The findings provide a foundation for the treatment of HF by FZYX.

## 2 Materials and methods

### 2.1 Gene chip analysis and identification of differentially expressed genes based on the gene expression omnibus

Firstly, we retrieved HF gene expression profiles and selected “Homo sapiens” entries from the gene expression omnibus (GEO) database. After reading the abstract, we selected entries according to the following criteria: 1) The number of samples was >40 (>20 for both experimental and control groups); 2) Healthy people were used as the control group; 3) Clinical samples were myocardial tissue. Secondly, Matrix and Platforms were downloaded after targets were selected, and the R packages BiocManager, Limma and Pheatmap were used to perform a secondary analysis of the chip data, with |log_2_FC (fold change) | > 0.5 and adjPvalue < 0.05 as the cut-off criteria for differentially expressed genes (DEGs). Finally, we selected the top 20 genes with the highest up- and downregulation to draw a heatmap. Genes unaffected and differential genes were used to draw a volcano map.

### 2.2 Fuzheng Yangxin Recipe active ingredient screening and target prediction

The BATMAN-TCM database (Liu et al., 2016) and the ETCM database (Xu et al., 2019) were used to screen effective ingredients for each TCM concoction, with adjusted *p*-value = 0.05 and score cutoff = 20 when using the BATMAN-TCM database, and Druglikeness Grading = ‘Moderate’ or ‘Good’ when using the ETCM database. The obtained components were used by the SwissTargetPrediction database (http://www.swisstargetprediction.ch/) to predict targets, which were organised into a table according to herb-ingredient code-ingredient name-target.

### 2.3 Construction of a traditional Chinese medicine compound regulatory network

DEGs in Section 2.1 and target genes of FZYX identified in Section 2.2 were used to draw a Venn diagram, and Cytoscape (Version 3.8.2) was used to construct a regulatory network of herb components with the names, intersecting genes, and active ingredients of medicines as nodes (Shannon et al., 2003).

### 2.4 Construction of a protein-protein interaction network

Researchers used “BisoGenet” in Cytoscape and data from six major human PPI databases (BioGRID, BIND, MINT, HPRD, DIP and intAct) to perform PPI network analysis of overlapping genes (Martin et al., 2010). The CytoNCA plug-in was used to carry out network topology analysis and to set degree centrality (DC) ≥20% and between centrality (BC) ≥20% as the core node screening conditions. Key nodes were important proteins in drug-target-gene information transmission.

### 2.5 Functional enrichment analysis

Gene Ontology (GO) provides functional annotation for biological process (BP), cellular component (CC) and molecular function (MF) categories. The Kyoto Encyclopedia of Genes and Genomes (KEGG) database was applied to analyse the signalling pathways in which the differential genes are involved. The R packages org.Hs.eg.db, DOSE, clusterProfiler, enrichplot, colorspace, stringi and ggplot2 can be used for visualisation of GO and KEGG results. According to the value and significance of enrichment (*p*-value cut-off <0.05, q-value cut-off <0.05), a bubble diagram was generated, KEGG pathways were imported into Cytoscape, and a pathway-gene network was drawn based on degree values.

### 2.6 Molecular docking

The intersection of key nodes in the PPI network and high relevance genes in pathway-gene network were selected as candidate genes for molecular docking. The receptors 3D format of the core targets were downloaded from the Protein Data Bank (https://www.rcsb.org/) (Burley et al., 2021), and operations including dehydration, hydrogenation and ligand extraction were performed using Pymol 2.5 software (Mooers, 2020). Autodock Vina 1.1.2 software was used for molecular docking (Trott and Olson, 2010).

### 2.7 Medicine preparation

*Panax notoginseng (Burkill) F. H. Chen* (60100000994), *Panax ginseng C. A. Meyer* (60100000951), *Rhodiola crenulata (Hook.f.et Thoms.) H. Ohba* (SA200226005), *Aconitum carmichaeli Debx* (SA200325013), *Ophiopogon japonicus (Linn. f.) Ker-Gawl* (60100000925), *Astragalus membranaceus (Fisch.) Bunge* (60100000921), *Angelica sinensis (Oliv.) Diels* (SA200530014) and *Rehmannia glutinosa Libosch* (60100000919) were purchased from China Resources Sanjiu (Beijing, China). In addition, angiotensin receptor neprilysin inhibitors (ARNI) were purchased from Novartis (SDC563, Beijing, China) and employed as positive control drugs.

### 2.8 Establishment of an myocardial infarction rat model and groupings

This experiment included 40 specific pathogen free male Sprague Dawley rats weighing 200 ± 20 g purchased from Charles River (SYXK 2018-0018, Beijing, China,). Animals were raised in the experimental animal room of Xiyuan Hospital. Standard synthetic feed was provided with free access to drinking pure water, the temperature was (24 ± 2°C), the humidity was 45%–50%, and a 12 h/12 h light/dark photoperiod was employed. Animal experiments were reviewed and approved by the ethics committee of Xiyuan Hospital. Rats were randomly divided into four groups; a sham operation group, a model group, an FZYX group (4.2 g/kg) and an ARNI (68 mg/kg) group. Animal drug dosage was based on the equivalent conversion between body surface area and weight of animals and humans (Huang et al., 2004), as well as previous related studies (Pfau et al., 2019; Vaskova et al., 2020). Rats in the sham operation and model groups were intragastric with equal amounts of phosphate-buffered saline (PBS). The drug was administered continuously for 42 days (Gan et al., 2014; Sunagawa et al., 2014; Wang et al., 2020).

MI-induced HF model rats were established by ligating the left anterior descending coronary artery. Briefly, rates were intubated following being anesthetised with 1% sodium pentobarbital (50 mg/kg intraperitoneal injection) using an ALC-V8S-P small animal ventilator (Alcott Biotech, Shanghai, China) at a rate of 70 breaths per min, ribs were separated to expose the heart, and a 4/0 medical silk thread was employed to ligate the artery 2–3 mm below the left atrial appendage. If the electrocardiogram indicated ST-segment elevation and the colour of the ligation area was immediately pale, ligation was considered successful (Lindsey et al., 2018). In the sham operation group, rats were threaded but arterial ligation was not performed.

### 2.9 Echocardiography assessment

After rats were fed drugs for 42 days, uninformed researchers anesthetized them with 1% sodium pentobarbital (50 mg/kg), and two-dimensional M-mode and B-mode echocardiography were performed using a Vevo2100 instrument (Visualsonics, Toronto, Canada) to assess left ventricular function. The left ventricular ejection fraction (LVEF), left ventricular fractional shortening (LVFS), left ventricular end-diastolic volume (LVEDV), left ventricular end-systolic volume (LVESV), left ventricular internal dimension end-diastolic (LVIDd) and left ventricular internal dimension end-systolic (LVIDs) were automatically calculated by the echocardiographic system.

### 2.10 Pathological section staining

Heart tissue of rats fed drugs for 42 days was collected and fixed in 4% paraformaldehyde overnight. Myocardial tissue was embedded and fixed in paraffin to prepare 4 μm sections. Different concentrations of ethanol were used for dewaxing, and samples were stained using a modified Masson’s Trichrome Stain Kit (G1346, Solarbio, Beijing, China) and a hematoxylin-eosin (HE) Staining Kit (G1120, Solarbio). Finally, myocardial tissue morphology and fibrosis degree were observed under a 400× microscope. Collagen volume fraction (CVF) was analyzed by Image-Pro Plus 6.0 (Media Cybernetics, Inc., Rockville, Maryland, United States). CVF = area of collagen/total area ×100%.

### 2.11 Enzyme-linked immunosorbent assay

Blood was collected from the abdominal aorta, incubated at room temperature for 1 h, then centrifuged at 1000 *g* for 10 min at 4°C. Enzyme-linked immunosorbent assay (ELISA) was employed to determine the level of N-terminal pro-B-type natriuretic peptide (NT-proBNP; AD2817Ra, Andygene, Beijing, China) according to the manufacturer’s instructions.

### 2.12 Western blotting

The infarction border area of the left ventricular myocardium was rapidly frozen in liquid nitrogen. The RIPA buffer (R0020, Solarbio) was added, the tissue was homogenised on ice, then centrifuged at a low temperature to extract total protein. Protein concentration was measured using a bicinchoninic acid (BCA) protein assay kit (AR1189, Boster, Wuhan, China). Equal amounts of proteins (50 μg) were separated based on molecular weight by 8%–12% sodium dodecyl sulphate polyacrylamide gel electrophoresis (SDS-PAGE), then transferred to a polyvinylidene fluoride (PVDF) membrane. Membranes were blocked with quick block buffer for western blot (P1623, Applygen, Beijing, China) at room temperature and incubated overnight at 4°C in the presence of primary antibody (Table 1). The membranes were washed three times with Tris Buffered Saline with Tween 20 (TBST), then incubated with the secondary antibody at room temperature for 1 h. The membrane was then developed using a western blotting 3,3′-diaminobenzidine (DAB) Chromogenic Assay Kit (SA2025, Boster), and bands were analysed using Image J software (Schneider et al., 2012).

**TABLE 1:** Antibodies used in western blotting.

Protein	Antibody	Concentration
BCL-2	Anti-BCL-2 (bs-20351R, Bioss)	1 μg/ml
BAX	Anti-BAX antibody (bs-4564R, Bioss)	1 μg/ml
Caspase-3	Anti-cleaved caspase-3 (ab2302, Abcam)	0.5 μg/ml
pSTAT3	Anti-phospho-STAT3 (Tyr705) (bs-1658R, Bioss)	2 μg/ml
STAT3	Anti-STAT3 (bs-20382R, Bioss)	2 μg/ml
GAPDH	Anti-GAPDH (bs-0755R, Bioss)	1 μg/ml
	Goat anti-rabbit IgG H&L/HRP second antibody (bs-40295G-HRP, Bioss)	2 μg/ml

### 2.13 Immunohistochemistry

Tissue sections were dewaxed and rehydrated, then subjected to antigen repair using the citric acid solution (pH 6.0). Hydrogen peroxide (3%) was used to block sections for 10 min, and 5% goat serum (SL038, Solarbio) was added and incubated for 1 h. Tissue sections were incubated with primary antibody (cleaved Caspase-3, 2 μg/ml, ab2302, Abcam, Cambridge, Massachusetts, United States) overnight at 4°C, followed by secondary antibody (3 μg/ml, bs-40295G-HRP, Bioss, Beijing, China). Finally, immunohistochemical reactions were analysed using a DAB Kit (AR1022, Boster) and nuclei were stained with HE (G1080, Solarbio). Images were observed under a microscope at 400× magnification. The integrated option density (IOD)/area was analysed using Image-Pro Plus 6.0.

### 2.14 Quantitative real-time PCR

RNA was extracted from the infarction border area of the left ventricular myocardium according to the instructions supplied with the Total RNA Extraction Kit (DP419, Tiangen, Beijing, China). The PrimeScript RT reagent Kit (RR037A, TaKaRa Bio, Beijing, China) was used for reverse transcription of RNA samples into cDNA. Primer sequences are listed (Table 2). Each 20 μl contained 6.8 μl of diethyl pyrocarbonate (DEPC) water, 100 μl of THUNDERBIRD SYBR qPCR Mix (QPS-201, Toyobo, Shanghai, China), 0.6 μl of forward primer, 0.6 μl of reverse primer, and 2 μl of cDNA. Thermal cycling involved an initial denaturation step at 95°C for 10 min, followed by 40 cycles at 95°C for 10 s, 55–60°C for 30 s, and 72°C for 10 s. The expression level of each gene was calculated using the 2^−ΔΔCT^ method (Chen et al., 2021).

**TABLE 2:** Primer sequences for each gene.

Gene	Primer sequence (5′→3′)	Temp (°C)
BCL-2	Forward: GGT​GAA​CTG​GGG​GAG​GAT​TG	Forward: 60.03
	Reverse: AGA​GCG​ATG​TTG​TCC​ACC​AG	Reverse: 60.02
BAX	Forward: CACGTCTGCGGGGAGTCA	Forward: 61.68
	Reverse: TAG​GAA​AGG​AGG​CCA​TCC​CA	Reverse: 59.95
Caspase-3	Forward: GAG​CTT​GGA​ACG​CGA​AGA​AA	Forward: 59.13
	Reverse: TAA​CCG​GGT​GCG​GTA​GAG​TA	Reverse: 60.03
STAT3	Forward: CTG​AGG​TAC​AAT​CCC​GCT​CG	Forward: 60.25
	Reverse: TCG​GTC​AGT​GTC​TTC​TGC​AC	Reverse: 59.97
GAPDH	Forward: GCA​TCT​TCT​TGT​GCA​GTG​CC	Forward: 60.11
	Reverse: GAT​GGT​GAT​GGG​TTT​CCC​GT	Reverse: 60.03

### 2.15 Statistical analysis

Data were statistically analysed by SPSS Statistics software (Version 25, IBM Corp., Armonk, New York, United States). All data are presented as mean ± standard deviation. One-way analysis of variance (ANOVA) with a completely random design was used for comparison between multiple groups, and the least significant difference (LSD) test was used for pairwise comparison between groups. When applying one-way ANOVA was not appropriate, the Kruskal-Wallis rank sum test was used. A *p*-value <0.05 was considered statistically significant.

## 3 Results

### 3.1 Differentially expressed genes analysis of heart failure based on the gene expression omnibus database

**FIGURE 1:**
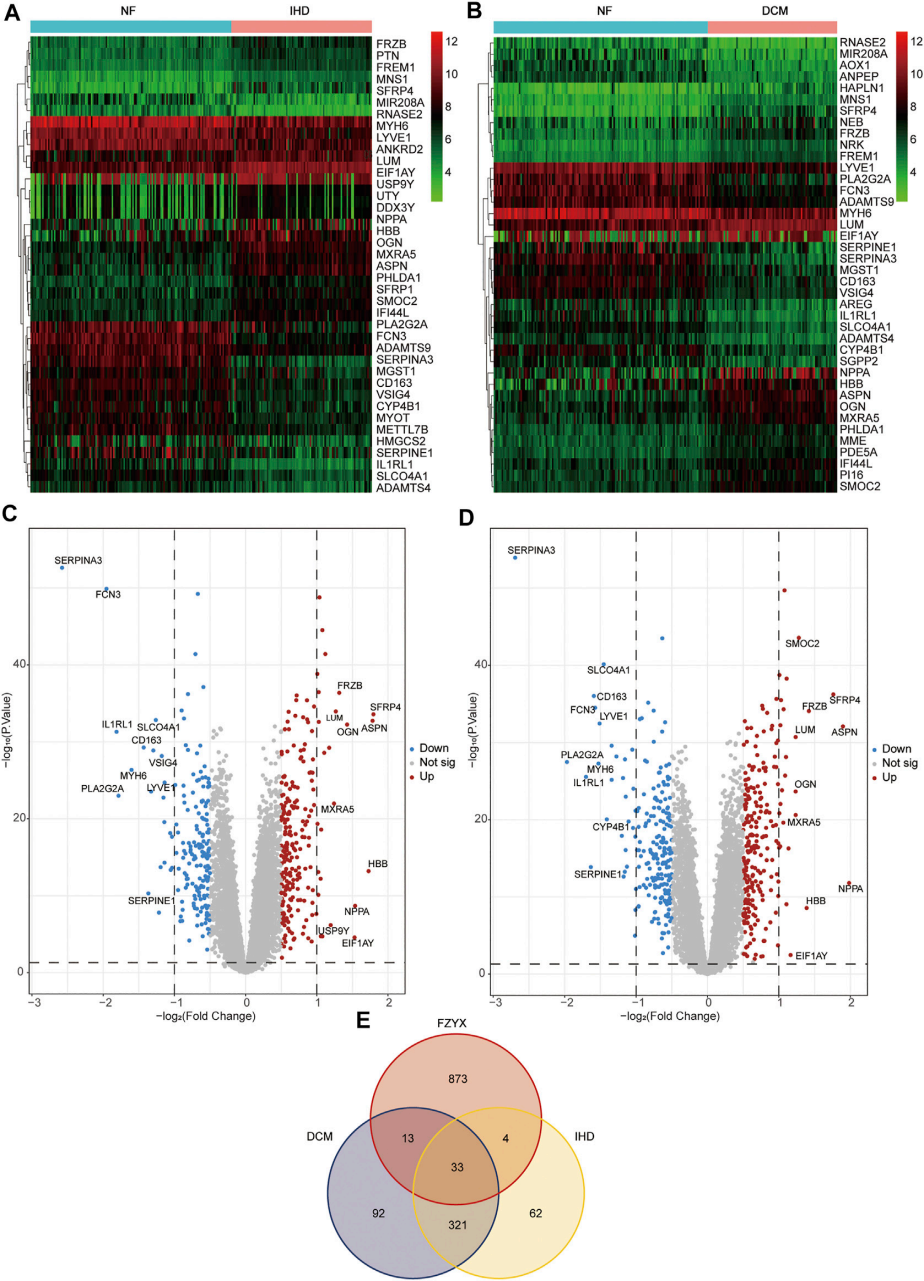
Differential gene acquisition and network construction. **(A)** Heatmap of non-failing (NF) VS ischemic heart disease (IHD). Green represents low expression, black represents medium expression, and red represents high expression. **(B)** Heatmap of NF VS idiopathic dilated cardiomyopathy (DCM). Green represents low expression, black represents medium expression, and red represents high expression. **(C)** Volcano map of IHD. **(D)** Volcano map of DCM. **(E)** Venn diagram of FZYX, IHD and DCM.

The GSE57338 chip and platform files from the GEO database were downloaded to obtain sample data from 136 non-failing (NF), 95 IHD with HF and 82 idiopathic dilated cardiomyopathy (DCM) with HF. To gain a better understanding of the different HF subtypes, we performed differential expression analysis on all types of HF patients based on the data. The patients’ characteristics (age and sex) were shown in Table 3. R software and related packages were used to filter and standardise the data and screen DEGs. In total, 420 DEGs between NF and IHD (238 upregulated and 182 downregulated) were identified, and the top 20 genes with the most significant upregulation and downregulation were plotted in a heatmap and the top 10 genes with the most significant upregulation and downregulation were marked in a volcano map (Figure 1A). 459 DEGs between NF and DCM (248 upregulated and 211 downregulated) were identified, and the top 20 genes with the most significant upregulation and downregulation were plotted in a heatmap and the top 10 genes with the most significant upregulation and downregulation were marked in a volcano map (Figure 1B). USP9Y and VSIG4 were found to be specifically expressed in IHD, while SMOC2 and CYP4B1 were discovered to be uniquely expressed in DCM. (Figures 1C–E). The DEGs are available in Supplementary Table S1.

**TABLE 3:** Characteristics of patients.

Characteristics	IHD, *n* = 95	DCM, *n* = 82	Non-failing, *n* = 136
Gender, No. (%)			
Male	81 (85.26%)	63 (76.83%)	73 (53.68%)
Female	14 (14.74%)	19 (23.17%)	63 (46.32%)
Age			
Years (mean ± SD)	59.11 ± 7.39	51.16 ± 13.98	49.36 ± 15.00

### 3.2 Fuzheng Yangxin Recipe components and targets

Components were identified by database screening, including 23 ingredients in *Panax notoginseng (Burkill) F. H. Chen*, 43 in *Panax ginseng C. A. Meyer*, 4 in *Rhodiola crenulata (Hook.f.et Thoms.) H. Ohba*, 8 in *Aconitum carmichaeli Debx*, 27 in *Ophiopogon japonicus (Linn. f.) Ker-Gawl*, 2 in *Astragalus membranaceus (Fisch.) Bunge*, 47 in *Angelica sinensis (Oliv.) Diels* and 4 in *Rehmannia glutinosa Libosch*. After deduplication, 405 components were identified in FZYX. In addition, targets were identified by database screening, including 369 ingredients in *Panax notoginseng (Burkill) F. H. Chen*, 423 in *Panax ginseng C. A. Meyer*, 124 in *Rhodiola crenulata (Hook.f.et Thoms. H. Ohba*, 44 in *Aconitum carmichaeli Debx*, 473 in *Ophiopogon japonicus (Linn. f.) Ker-Gawl*, 180 in *Astragalus membranaceus (Fisch.) Bunge*, 591 in *Angelica sinensis (Oliv.) Diels* and 77 in *Rehmannia glutinosa Libosch*. After deduplication, 923 targets in FZYX were identified (Supplementary Table S2).

### 3.3 Drug-disease intersection genes and herbal compound regulatory network analysis

**FIGURE 2:**
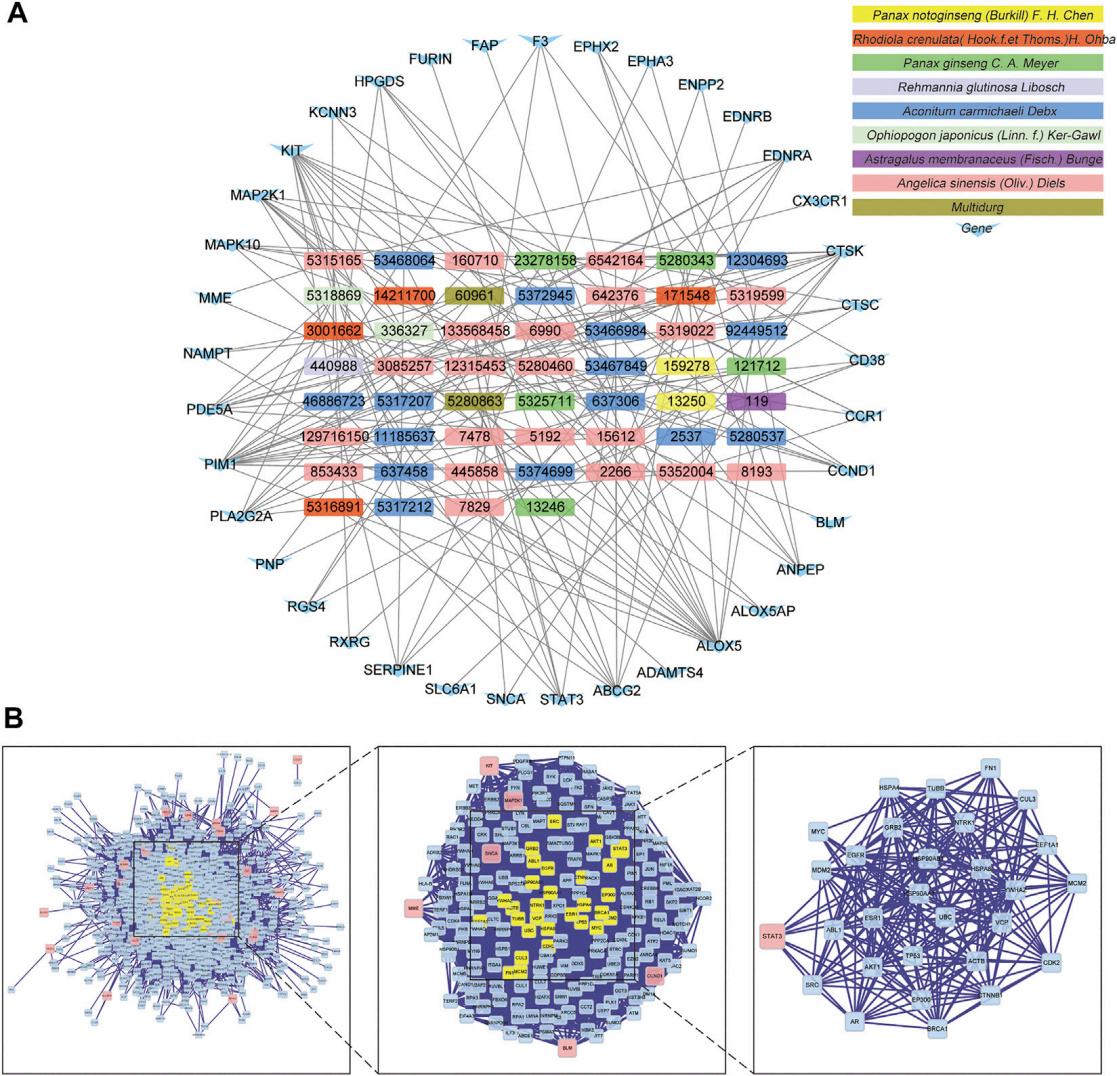
Regulatory network and PPI network. **(A)** Herb-ingredients-targets network. **(B)** PPI network and its topological analysis. The initial PPI network of 37 intersection genes had 1,260 nodes and 18,091 edges, whereas the final network included 29 nodes and 243 edges.

Target genes of FZYX were intersected with disease-related genes, and 37 intersection genes were obtained (Figure 1E). These intersection genes are potential targets of FZYX in the treatment of IHD-induced HF. An ingredient-intersection gene regulatory network diagram of FZYX was constructed with Cytoscape 3.8.2 software, containing 90 nodes (37 genes, 53 ingredients) and 182 edges (Figure 2A). Links between nodes represent the functional relationships of these nodes, and the greater the number of connected nodes, the more important the role of the target or ingredient in the network. Analyse-Network was used to calculate the network degree, and ingredients with higher degrees included Gomisin A, Kumatakenin, Methylophiopogonanone B and Senkyunolide A (Supplementary Table S3).

### 3.4 Topological analysis of the protein-protein interaction network

Researchers used BisoGenet to construct a PPI network of 37 intersection genes, which includes 1,260 nodes and 18,091 edges (Supplementary Table S4); the final 29 key nodes and 243 edges were obtained by screening with the CytoNCA software package. Based on the core network, signal transducer and activator of transcription 3 (STAT3) was identified as the key protein in the PPI core network (Figure 2B).

### 3.5 Gene ontology functional analysis

**FIGURE 3:**
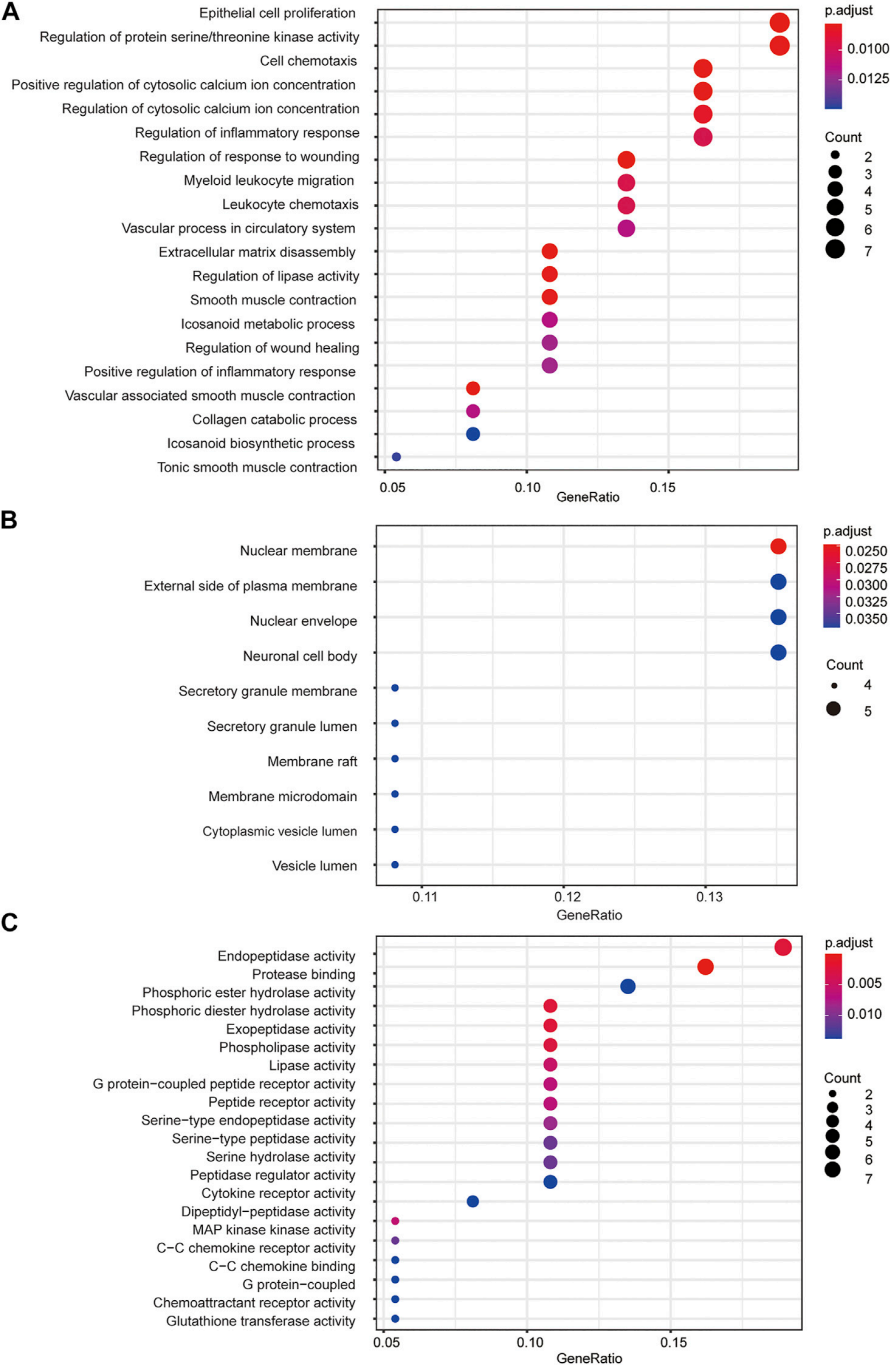
GO functional analysis. **(A)** GO biological processes of 37 core genes. **(B)** GO cellular component of 37 core genes. **(C)** GO molecular function f 37 core genes.

To further understand the mechanism of FZYX, GO and KEGG were performed. GO functional analysis was performed on 37 key intersection genes with *p* < 0.05. The final enrichment results comprised 1) 146 BP subcategories including vascular-associated smooth muscle contraction, extracellular matrix disassembly, positive regulation of cytosolic calcium ion concentration, regulation of lipase activity, regulation of protein serine/threonine kinase activity, regulation of inflammatory response, and collagen catabolic process; 2) 10 CC subcategories including nuclear membrane, external side of plasma membrane and nuclear envelope; 3) 40 MF subcategories including protease binding, endopeptidase activity, phosphoric diester hydrolase activity, exopeptidase activity and phospholipase activity. The top 20 BP and MF subcategories and the top 10 CC subcategories are shown in Figure 3. Details are provided in Supplementary Table S5.

### 3.6 Kyoto encyclopedia of genes and genomes functional analysis

**FIGURE 4:**
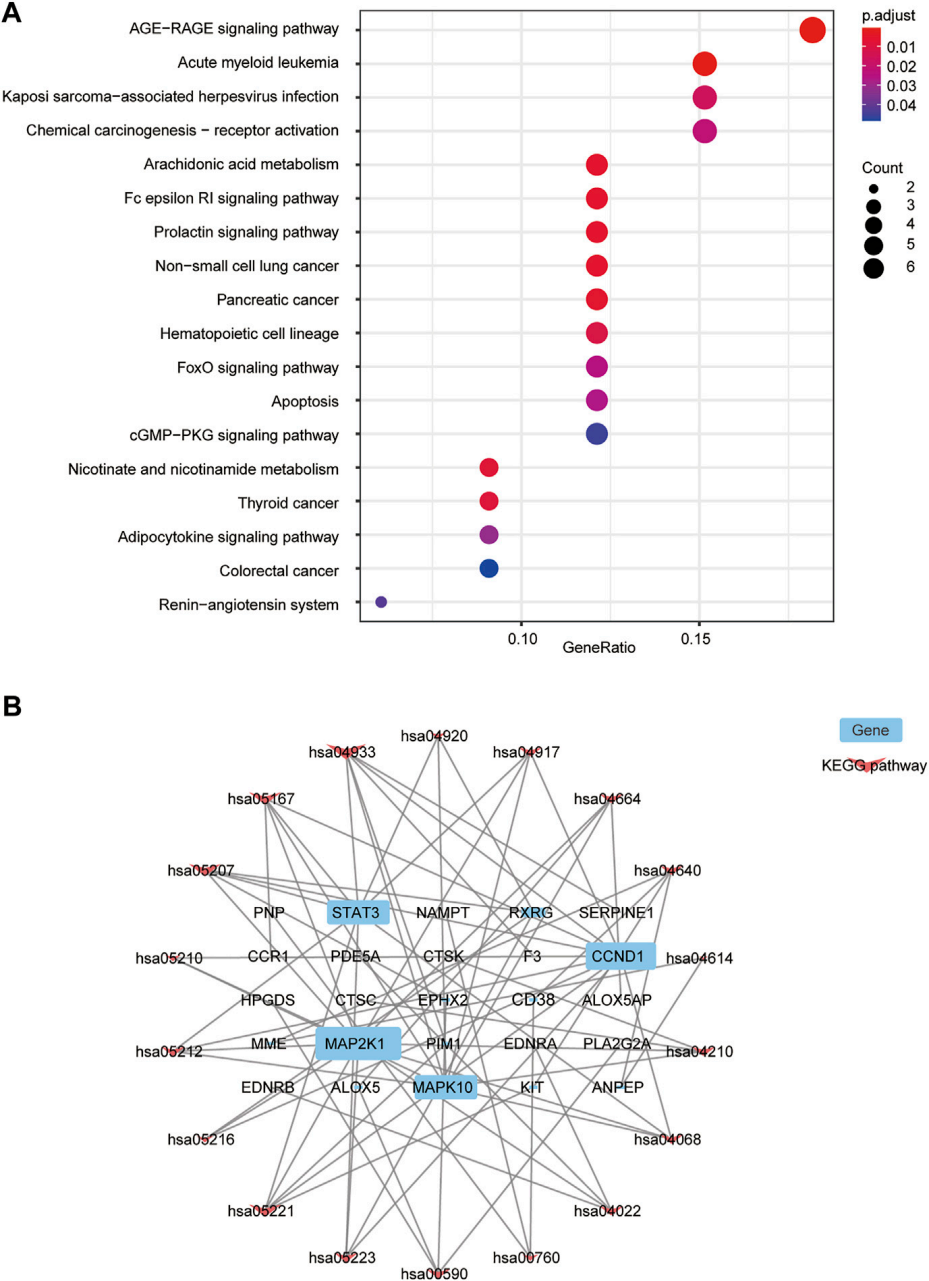
KEGG functional analysis. **(A)** KEGG bubble diagram. **(B)** Interaction network of 25 targets (blue) and 18 pathways (pink) to indicate pathways-targets network.

KEGG pathway analysis showed that 18 signal pathways were enriched (Figure 4A). Based on previous work on the HF mechanism, our results suggest that FZYX may act in HF through the advanced glycation end-product (AGE)-receptor for AGE (RAGE) signalling pathway, arachidonic acid metabolism, the high-affinity receptors for immunoglobulin E (Fc epsilon RI) signalling pathway, the prolactin signalling pathway, nicotinate and nicotinamide metabolism, the forkhead box O (FOXO) signalling pathway, apoptosis, the adipocytokine signalling pathway, the renin-angiotensin system, and the cyclic guanosine monophosphate (cGMP)- protein kinase G (PKG) signalling pathway. Cytoscape was used to construct a “Gene-KEGG signalling pathway” network. The results showed that mitogen-activated protein kinase kinase 1 (MAP2K1), STAT3, cyclin D1 (CCND1) and mitogen-activated protein kinase 10 (MAPK10) were the most enriched genes, suggesting they might be related to the mechanism of FZYX (Figure 4B). Details are provided in Supplementary Table S6.

### 3.7 Molecular docking

**FIGURE 5:**
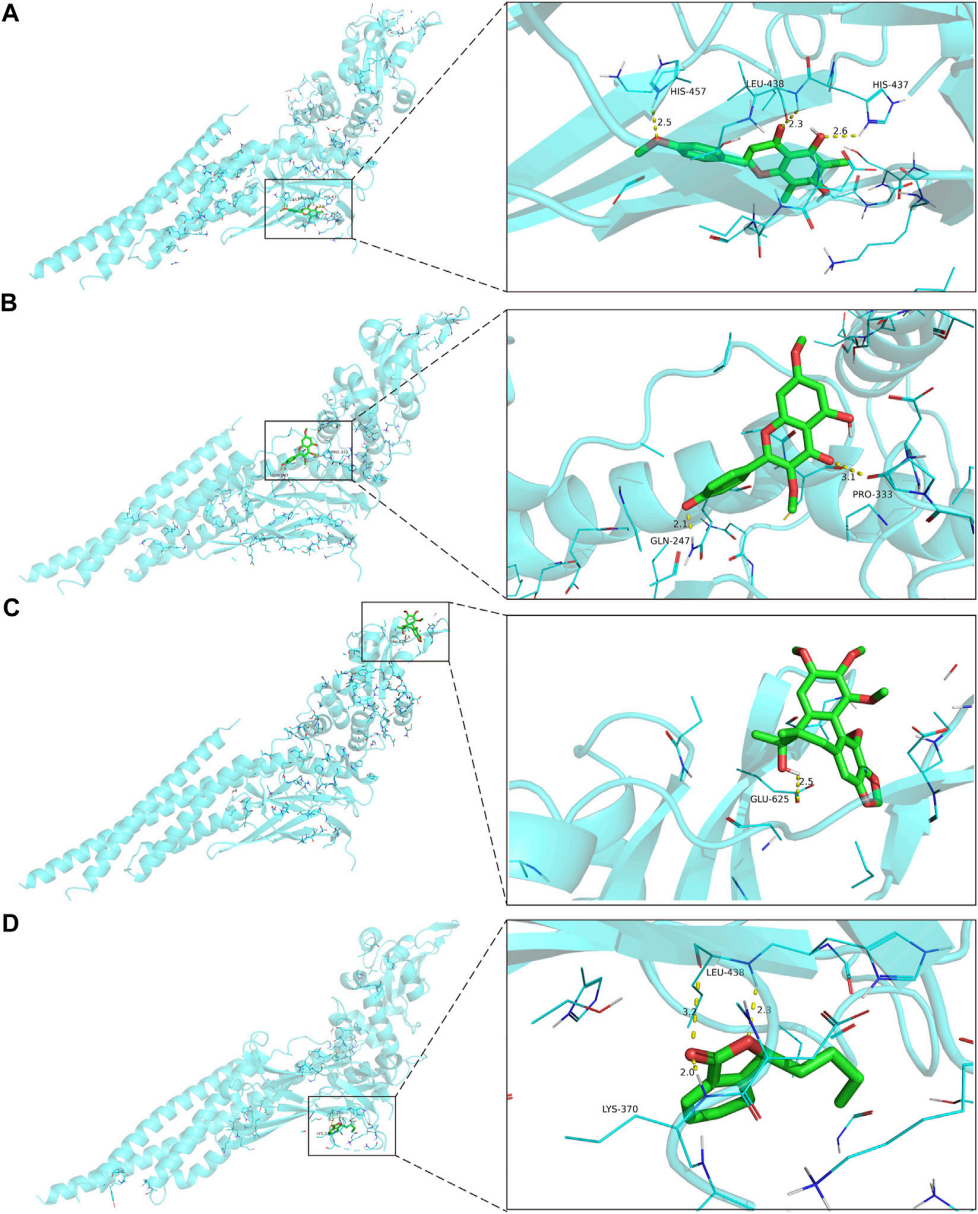
The binding site of the main active components of FZYX and STAT3. **(A)** Gomisin A and STAT3. **(B)** Kumatakenin and STAT3. **(C)** Methylophiopogonanone B and STAT3. **(D)** Senkyunolide A and STAT3. The dashed line in the figure is the hydrogen bond and the value is the bond length.

Molecular docking was performed with the main compounds of FZYX and the intersection targets, namely STAT3. The lower the affinity value in the docking findings, the more stable the contact between the targets and the active component was. Through molecular docking, it was found that STAT3 had good binding activities with the active components of FZYX (Table 4). These compounds bind to STAT3 through interacting with various amino acid residues, such as HIS-457, LEU-438, HIS-437, GLN-247, PRO-333, GLU-625, LEU-438 and LYS-370. The binding interactions and the binding sites of compounds-targets were shown in Figure 5. These results suggest that FZYX may act through STAT3.

**TABLE 4:** Details of molecular docking.

Component	Structure	PubChem CID	Target	PDB ID	Binding energy (kcal/mol)
Methylophiopogonanone B	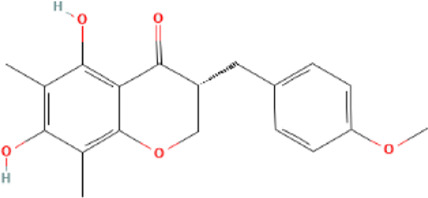	46886723	STAT3	6NJS	−7.4
Gomisin A	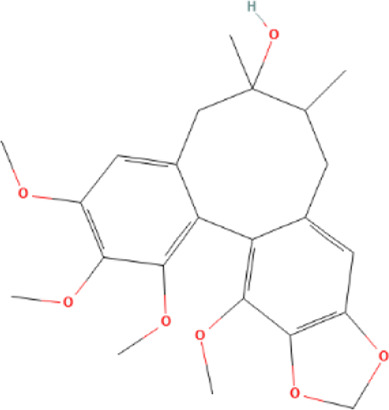	634470	STAT3	6NJS	−6.3
Kumatakenin	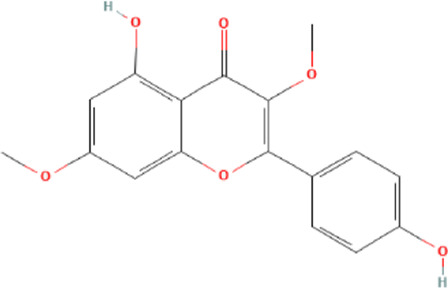	5318869	STAT3	6NJS	−6.7
Senkyunolide A	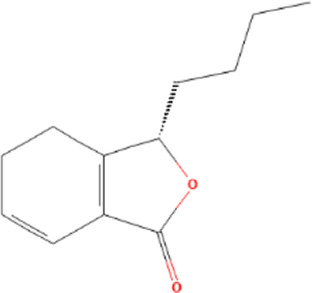	3085257	STAT3	6NJS	−5.6

### 3.8 Effects of Fuzheng Yangxin Recipe on cardiac function and structural alterations in heart failure model rats

**FIGURE 6:**
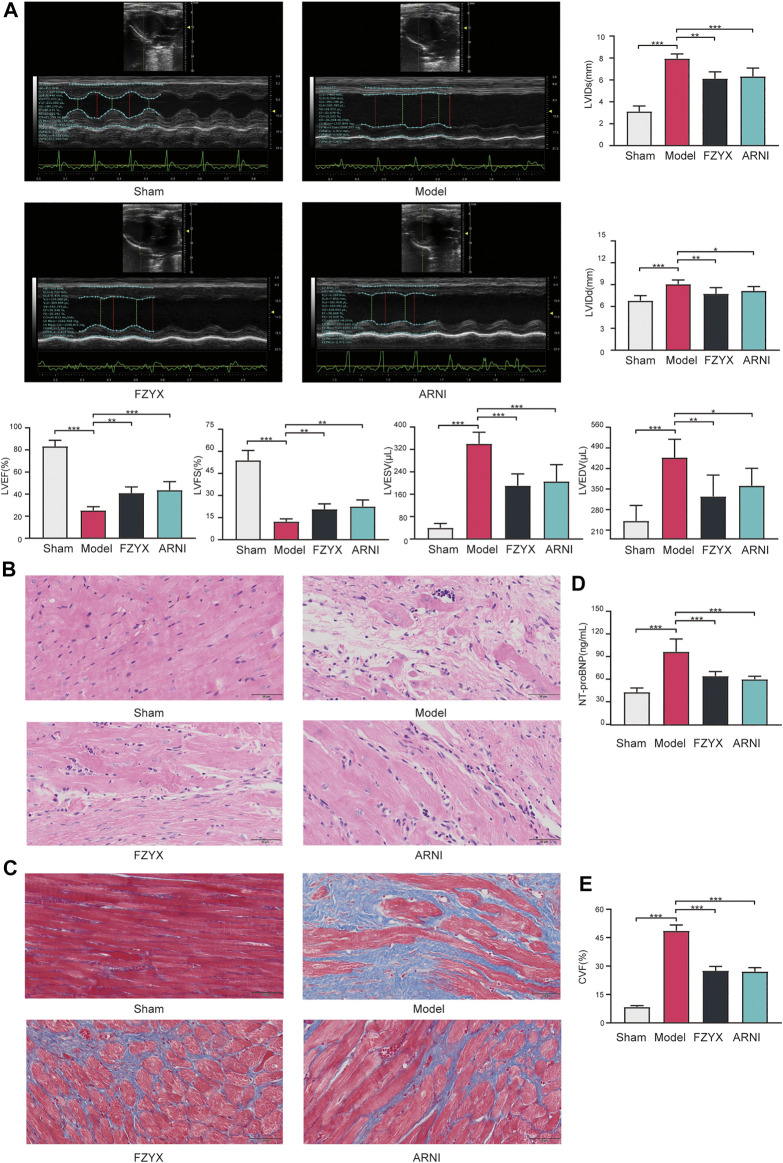
Effects of FZYX on cardiac function and structural alterations in HF model rats. **(A)** Echocardiographic results (*n* = 5). Statistical analysis was performed including the LVEF, LVFS, LVESV, LVEDV, LVIDs and LVIDd. **(B)** HE staining. **(C)** Masson’s staining. **(D)** NT-proBNP (*n* = 5). **(E)** CVF (*n* = 5) *p < 0.05, **p < 0.01, ***p < 0.001 vs. controls.

At the end of the experiment cycle, two animals in the model group, one in the FZYX group and none in the remaining groups died. Echocardiographic results showed that LVEF and LVFS were decreased and LVIDs, LVIDd, LVESV and LVEDV were increased in the HF model group compared with the sham operation group, suggesting that cardiac structure and function were severely impaired after acute MI (Figure 6A). LVEF and LVFS were significantly increased and LVIDs, LVIDd, LVESV and LVEDV were significantly decreased after treatment with FZYX (Figure 6A). The HE staining results showed that myocytes in the sham operation group were structurally intact and well-arranged, while myocytes in the model group were structurally disorganised and loosely arranged. In addition, nuclei in the model group underwent consolidation or fragmentation, and obvious inflammatory infiltration was observed (Figure 6B). A blue collagen fiber distribution in the interstitial space of myocardial cells was evident in the model group following Masson’s staining (Figure 6C), and treatment with FZYX significantly improved these pathological changes and CVF significantly decreased (Figure 6E). NT-proBNP is a diagnostic marker of HF(McDonagh et al., 2021). Our results showed that serum NT-proBNP levels were significantly increased in the model group compared with the sham operation group (Figure 6D). After FZYX treatment, all the above indices were significantly decreased, suggesting that FZYX protected myocardial structure and cardiac function. The effects of ARNI were similar to those of FZYX.

### 3.9 Regulation of apoptosis and signal transducer and activator of transcription 3 by Fuzheng Yangxin Recipe

**FIGURE 7:**
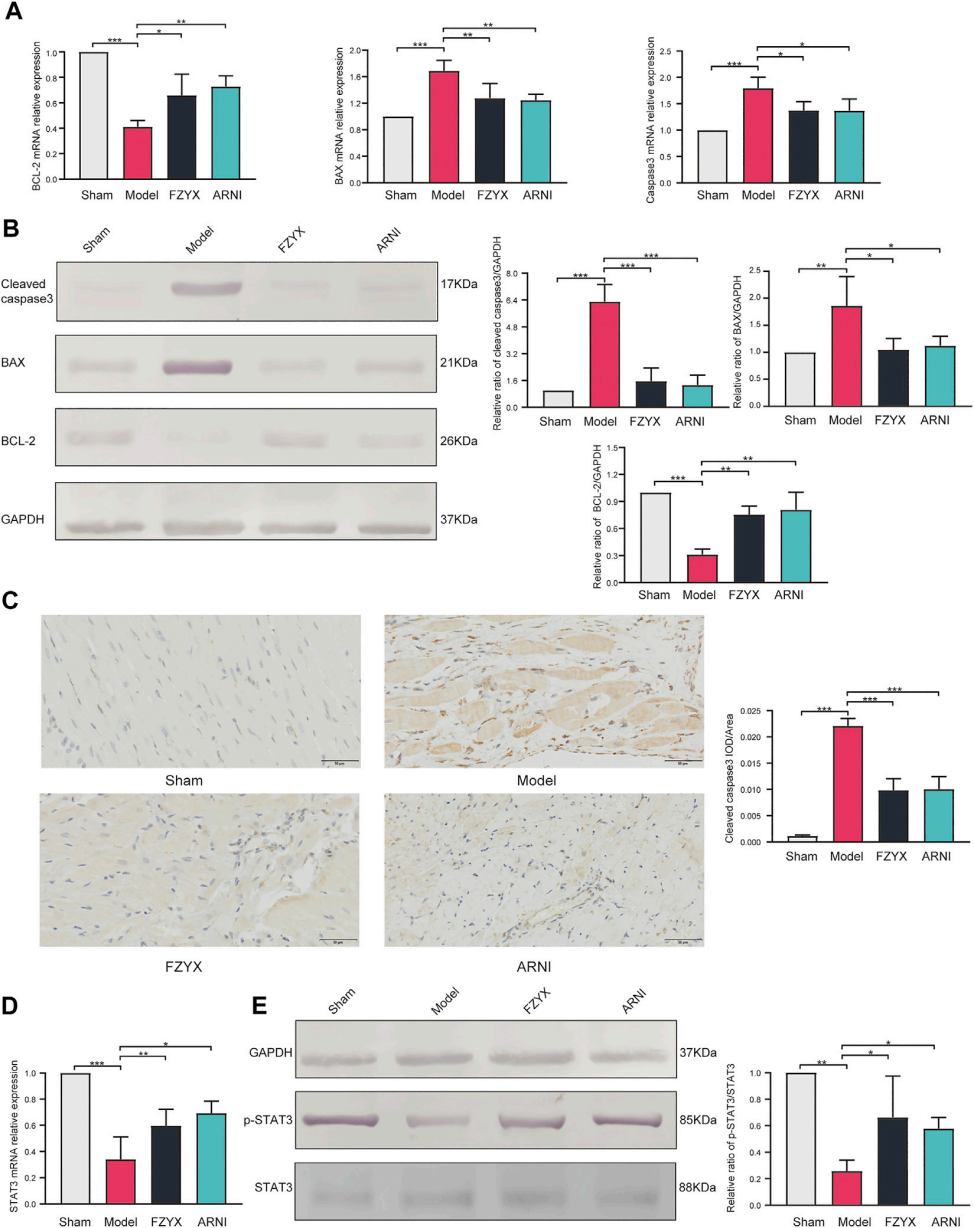
Regulation of apoptosis and STAT3 by FZYX. **(A)** mRNA expression levels of BCL-2, BAX and Caspase-3 (*n* = 3). **(B)** Protein abundance of BCL-2, BAX and Caspase-3 (*n* = 3). **(C)** immunohistochemical analysis of Caspase-3 (*n* = 5). **(D)** mRNA expression levels of STAT3 (*n* = 3). **(E)** Protein abundance of pSTAT3 and STAT3 (*n* = 3). *p < 0.05, **p < 0.01, ***p < 0.001 vs. controls.

To verify the underlying mechanism of the network pharmacological findings, expression of apoptosis-related genes at mRNA and protein levels in cardiomyocytes was measured by Quantitative real-time PCR (qRT-PCR) and western blotting, respectively (Figures 7A,B). The results showed that compared with the sham operation group, expression of anti-apoptotic B cell lymphoma-2 (BCL-2), pro-apoptotic BCL-2 associated X (BAX) and Caspase-3 was significantly increased in the model group. FZYX upregulated the expression of BCL-2 and decreased the expression of BAX and Caspase-3 in HF. Immunohistochemical analysis of Caspase-3 protein was consistent with the above, suggesting that FZYX attenuated cardiac remodelling after MI in rats by inhibiting apoptosis (Figure 7C).

The mRNA expression levels of STAT3 were measured by qRT-PCR, and the abundance of phosphorylated STAT3 and total STAT3 proteins was measured by western blotting according to the results of network pharmacology (Figures 7D,E). Compared with the sham operation group, STAT3 mRNA expression levels were reduced and the pSTAT3/STAT3 ratio was decreased in the model group, while FZYX upregulated STAT3 mRNA levels and protein phosphorylation.

## 4 Discussion

Based on pattern identification and disease, TCM uses various herb combinations according to conditions to achieve the goal of holistic treatment. Therefore, TCM prescriptions often contain numerous herbs, resulting in complex chemical composition and numerous targets. Hence, modern research faces great challenges in deciphering their mechanisms and modes of action. Network pharmacology retrieves known chemical components of herbs in prescriptions based on a variety of databases and analysis software. From the data, targets of the chemical components, sites of action, molecules, and pathways can be obtained, allowing comprehensive analysis of the mechanisms of the compounds, and providing a reference for further clinical research on a specific mechanism (Li and Zhang, 2013).

The present study was divided into two parts: network pharmacology and experimental validation. We first used GSE57338 to identify DEGs in HF. GSE57338 includes three groups: NF, IHD and DCM. V-set and immunoglobulin domain containing 4(VSIG4) is specifically expressed in tissue-resident macrophages and can mediate various cellular events. Inhibition of the expression of VSIG4 can significantly increase the secretion of cytokines in macrophages and aggravate the inflammatory response^[19, 20]^. This suggests a strong relationship between inflammation and IHD, although further validation is needed considering that VSIG4 has been poorly studied in HF. Extracellular matrix changes play an important role in the development of DCM. Different from fibrosis triggered by ischemic conditions, DCM manifests as diffuse fibrosis^[21]^. Secreted modular calcium-binding protein 2(SMOC2), as a matrix protein, is a non-structural component of the extracellular matrix^[22]^. Whether SMOC2 affects extracellular mechanism components in DCM and plays a key role also needs to be further verified

FZXY has a good clinical effect on IHD-induced HF, but its specific active components are still unclear. We used the database to collect and screen the active components of FZYX, and finally obtained 143 compounds. Some of these ingredients exert their cardioprotective effects through known mechanisms determined by *in vitro* and *in vivo* experiments. Kaempferol, a natural flavonoid compound, possesses anti-inflammatory and antioxidant activities (Devi et al., 2015; Yeung et al., 2019). On the one hand, Kaempferol can stimulate the notch receptor 1 (Notch1)/phosphatase and tensin homolog (PTEN)/protein kinase B (AKT) signalling pathway, activate sirtuin 1 (SIRT1), inhibit the MAPK signalling pathway to reduce myocardial ischemic injury (Guo et al., 2015; Suchal et al., 2016; Huang and Qi, 2020); on the other hand, it can inhibit cardiac hypertrophy via regulation of the apoptosis signal-regulating kinase1 (ASK1)/MAPK signalling pathway (Feng et al., 2017) and modulation of the nuclear factor-kappaB (NF-κB)/MAPK signalling pathway to inhibit ventricular fibrosis (Du et al., 2019). Salidroside is the main active ingredient of *Rhodiola crenulata (Hook.f.et Thoms.) H. Ohba*, and has good biological activity for the treatment of cardiovascular and metabolic diseases (Zhao et al., 2021). Salidroside can modulate disordered homeostasis of energy and lipid metabolism to fight against hypoxia damage in cardiomyocytes (Liao et al., 2020). In addition, salidroside attenuates the pathological process of myocardial remodelling in mice with MI by downregulating the expression levels of tumor necrosis factor-alpha (TNF-α), transforming growth factor- beta 1 (TGF-β1), interleukin-1beta (IL-1β) and BAX, and upregulating the expression of BCL-2, vascular endothelial growth factor (VEGF), AKT and endothelial nitric oxide synthase (eNOS) (Chen et al., 2019).Ferulic acid, a phenolic compound with natural antioxidant activity, is able to improve cardiovascular functions and inhibit the pathogenetic cardiovascular disease process (Cheng et al., 2016). Clinical studies have found that ferulic acid is the main active ingredient in the treatment of coronary heart disease (Li et al., 2013). In rat HF models, ferulic acid reduces oxidative stress and inhibits cardiomyocyte apoptosis by activating the nuclear factor erythroid 2-related factor 2 (NRF2) signalling pathway (Zhang et al., 2021).Butylidenephthalide is the main active ingredient of Angelica sinensis (Oliv.) Diels (Ying et al., 2013), which can attenuate cardiac fibrosis by regulating the phosphatidylinositol 3-kinase (PI3K)/STAT3-mediated macrophage phenotype in aged rats after MI(Lin et al., 2019). Quercetin (a natural polyphenolic compound) and its metabolites exert cardioprotective effects via antioxidant, anti-inflammatory and molecular pathway modulation in a wide range of experimental models of cardiac injury (Ferenczyova et al., 2020).Quercetin improves cardiomyocyte hypoxic injury by regulating SIRT1/transmembrane BAX inhibitor motif containing 6 (TMBIM6)-related mitophagy and endoplasmic reticulum (ER) stress (Chang et al., 2021). In animal models, quercetin improves myocardial ischemia/reperfusion (I/R)-induced cardiomyocyte apoptosis via SIRT1/peroxisome proliferator-activated receptor gamma coactivator-1 alpha (PGC-1α) signalling (Tang et al., 2019). Quercetin can inhibit myocardial hypertrophy through glycogen synthase kinase-3 (GSK-3) (Chen et al., 2018) and inhibit myocardial fibrosis through the MAPK signalling pathway (Min et al., 2019). Ligustilide, a natural benzoquinone derivative, protects vascular endothelial cells and rescues high fat diet-induced atherosclerosis by activating multiple NRF2 downstream genes (Zhu et al., 2019).

After obtaining the active ingredients of FZYX, we used SwissTargetPrediction to predict the target of the drug. The results were intersected with the DEGs of IHD-induced HF, and 37 intersection genes were finally obtained. PPI and functional enrichment analyses were performed on these 37 genes to discover the underlying mechanisms. Based on the results of PPI and enrichment analysis, STAT3 and apoptosis were selected as the key roles of FZYX prescription for subsequent studies. The results of molecular docking also showed that the main components of FZYX and STAT3 had good binding activity. STAT3 can affect cell-to-cell communication, signal transduction and gene transcription. A large number of previous studies have confirmed the role of STAT3 in tumours (Tolomeo and Cascio, 2021). In recent years, many studies have found that STAT3 and its related signalling pathways also play a key regulatory role in the cardiovascular system, such as IHD, HF and myocardial hypertrophy (Hilfiker-Kleiner et al., 2005). Phosphorylation and activation of STAT3 have been widely observed in the heart after ischemia or Ischemia/Reperfusion I/R (Zhao et al., 2019; Takahashi et al., 2020). Western blotting was used to quantify and compare the phosphorylation of various proteins in pig left ventricular samples in a large number of I/R experiments, and the results showed that STAT3 is a common pathway for ischemic regulation (Kleinbongard et al., 2018). After subacute MI, downregulation and continuous impaired activation of STAT3 can lead to poor remodelling and HF (Hilfiker-Kleiner et al., 2010). One study infected myocardial-specific STAT3 knockout mice with tamoxifen for 14 consecutive days from the 11th day after MI to establish a subacute MI (STAT3 iCKO) mouse model. Results showed that the mortality of STAT3 iCKO mice was increased, myocardial fibrosis was significantly worsened, and heart function deteriorated (Enomoto et al., 2015). STAT3 also plays a role in macrophage-mediated post-MI repair, mainly through the IL10-STAT3-Galectin 3 axis promoting osteopontin-mediated generation of reparative macrophages, which promote MI by stimulating fibrosis and the clearance of apoptotic cells (Shirakawa et al., 2018). These results indicate that moderately active STAT3 in the heart endows resistance to cardiac remodelling during myocardial ischemia injury, and participates in the protective effect of various interventions on myocardial ischemia.

Apoptosis is the process of programmed cell death, which is primarily mediated by the death receptor pathway and the mitochondrial apoptosis pathway (Zhang et al., 2019). Apoptosis is an important cause of myocardial injury in patients with acute MI, and is involved in the subsequent development of ventricular remodelling and HF (Teringova and Tousek, 2017). Activation of STAT3 has been shown to reduce myocardial cell apoptosis during myocardial ischemia injury. STAT3-deficient mice have increased susceptibility to myocardial I/R injury and infarction, increased cardiac apoptosis, increased infarct size, and decreased cardiac functions and survival rate (Hilfiker-Kleiner et al., 2004). Activated STAT3 binds to cyclophilin D, a matrix protein that promotes mitochondrial permeability transition pore (MPTP) opening when bound to the mitochondrial inner membrane, and inhibits MPTP opening (Boengler et al., 2010). MPTP opening leads to the release of apoptotic factors, such as cytochrome C (Cyt C), into the cytoplasm (Cavalcante et al., 2019). Cyt C then interacts with apoptotic protease activating factor-1 (Apaf-1) and forms an apoptotic complex with the assistance of adenosine triphosphate (ATP) and deoxyadenosine triphosphate (dATP). The apoptotic complex recruits and activates Pro-Caspase-9 to form cleaved-Caspase-9, which activates Caspase-3 and Caspase-7, triggering the Caspase cascade reaction, finally leading to cell apoptosis (Zhu et al., 2021).

To test whether FZYX exerts cardiac protective effects through apoptosis and STAT3, we performed animal experiments. The results of echocardiography and NT-proBNP showed that FZYX could improve cardiac function in the HF model. The qRT-PCR and western blotting showed that FZYX could increase the expression of BCL-2 and decrease the expression of BAX, and Caspase-3. The results showed that FZYX had anti-apoptosis ability, which was consistent with the results of enrichment analysis. We also tested the effect of FZYX on STAT3 expression. The qRT-PCR showed that FZYX could upregulate STAT3 mRNA expression levels, and western blotting showed that the pSTAT3:STAT3 ratio was increased, indicating that the anti-apoptotic mechanism of FZYX might be related to STAT3.

## 5 Conclusion

In summary, this study systematically investigated the mechanism of FZYX in the treatment of HF through a combination of network pharmacology and experimental validation. Network pharmacology revealed that FZYX contains various active components that exert cardioprotective effects through multiple targets and signalling pathways. STAT3 may be the core factor of FZYX action, and the anti-apoptotic activity may be one of the most important effects of FZYX in protecting cardiac function. In addition, we verified the effect of FZYX on STAT3 expression and its anti-apoptotic effects in a rat model of ischemic HF.

## 6 Limitations

In this study, we only focused on the apoptotic pathway and STAT3, and did not fully utilize the results derived from network pharmacology. Second, this study is only a preliminary exploratory study, and the inhibitor group is not designed. In our subsequent study, a more reasonable design will be carried out to increase the reliability of the conclusion.

## Data Availability

The original contributions presented in the study are included in the article/Supplementary Material, further inquiries can be directed to the corresponding author.

## Glossary

AGE: Advanced glycation end-product
AKT: Protein kinase B
ANOVA: One-way analysis of variance
Apaf-1: Apoptotic protease activating factor-1
ARNI: Angiotensin receptor neprilysin inhibitors
ASK1: Apoptosis signal-regulating kinase1
ATP: Adenosine triphosphate
BAX: Pro-apoptotic BCL-2 associated X
BC: Between centrality
BCA: Bicinchoninic acid
BCL-2: B cell lymphoma-2
BP: Biological process
CC: Cellular component
CCND1: Cyclin D1
cGMP: Cyclic guanosine monophosphate
CVF: Collagen volume fraction
Cyt C: Cytochrome C
DAB: 3,3′-diaminobenzidine
dATP: Deoxyadenosine triphosphate
DC: Degree centrality
DCM: idiopathic dilated cardiomyopathy
DEGs: differentially expressed genes
DEPC: Diethyl pyrocarbonate
ELISA: Enzyme-linked immunosorbent assay
eNOS: Endothelial nitric oxide synthase
ER: Endoplasmic reticulum
FC: Fold change
Fc: epsilon RI, High-affinity receptors for immunoglobulin E
FOXO: Forkhead box O
FZYX: Fuzheng Yangxin Recipe
GEO: Gene expression omnibus
GO: Gene Ontology
GSK-3: Glycogen synthase kinase-3
HE: Hematoxylin-eosin
HF: Heart failure
I/R: Ischemia/reperfusion
IHD: Ischemic heart disease
IL-1β: Interleukin-1beta
IOD: Integrated option density
KEGG: Kyoto Encyclopedia of Genes and Genomes
LC: Liquid chromatography
LSD: Least significant difference
LVEDV: Left ventricular end-diastolic volume
LVEF: Left ventricular ejection fraction
LVESV: Left ventricular end-systolic volume
LVFS: Left ventricular fractional shortening
LVIDd: Left ventricular internal dimension end-diastolic
LVIDs: Left ventricular internal dimension end-systolic
MAP2K1: Mitogen-activated protein kinase kinase 1
MAPK: Mitogen-activated protein kinase
MF: Molecular function
MI: Myocardial infarction
MPTP: Mitochondrial permeability transition pore
MS: Mass spectrometry
NF: Non-failing
NF-κB: Nuclear factor-kappaB
Notch1: Notch receptor 1
NRF2: Nuclear factor erythroid 2-related factor 2
NT-proBNP: N-terminal pro-B-type natriuretic peptide
PGC-1α: Peroxisome proliferator-activated receptor gamma coactivator-1 alpha
PI3K: Phosphatidylinositol 3-kinase
PKG: Protein kinase G
PPI: Protein-protein interaction
PTEN: Phosphatase and tensin homolog
PVDF: Polyvinylidene fluoride
qRT-PCR: Quantitative real-time PCR
RAGE: Receptor for AGE
SDS-PAGE: Sodium dodecyl sulphate polyacrylamide gel electrophoresis
SIRT1: Sirtuin 1
STAT3: Signal transducer and activator of transcription 3
SMOC2: Secreted modular calcium-binding protein 2
TBST: Tris Buffered Saline with Tween 20
TCM: Traditional Chinese medicine
TGF-β1: Transforming growth factor- beta 1
TMBIM6: Transmembrane BAX inhibitor motif containing 6
TNF-α: Tumor necrosis factor-alpha
VEGF: Vascular endothelial growth factor
VSIG4: V-set and immunoglobulin domain containing 4
